# Vesico- Vaginal Fistula, with Laceration of the Anterior Lip of the Cervis Uteri, of Nearly Six Years Standing, Cured in Two Weeks

**Published:** 1855-12

**Authors:** N. Boseman

**Affiliations:** Montgomery


*Vesico-Vaginal Fistula, with Laceration of the Anterior Lip of the Cervis Uteri, of nearly six years standing, cured in two weeks.* By N. BOSEMAN, M. D. (Report read before the Sydenham Medical Society, of Montgomery, Ala.

The subject of this case was very kindly sent to my infirmary for treatment, by Dr. W. J Mitchell, of Tuskegee.

Delphia, (colored woman,) belonging to Dr. Robert Howard, of Macon county, was admitted on the 4th of April, aged 25; stout, well formed, and has the appearance of one enjoying good health; always menstruated regularly, etc. In August, 1850, she gave birth to her second and last child; has had no control over her urine ever since; was in labor nearly four days; delivery natural; child of medium size, strong and vigorous; did well, and is at present a large and healthy boy. Presentation was vertex, so far as can be ascertained. First labor, about a year before, lasted four days—the child was still-born, and of large size; delivery natural, recovery speedy, etc. In neither confinement was the patient attended by a physician.

Upon examination, I found the vagina to be very capacious—a circumstance of no little importance, as regards success, in any case, but especially is it so when the fistulous opening is found in close proximity to the cervix uteri. The injury sustained by the parts seemed, at first view, to be very extensive; but upon further inspection, they presented a more favorable appearance. In the anterior lip of the cervix was to be seen a deep cleft, presenting on either side, a considerable prominence ; at its anterior extremity was situated the fistula, oval in shape, and large enough to admit the index finger into the bladder. No effort seemed to have been made by nature to repair the cervical injury. In a case,* almost identical, which came under my observation last year a spontaneous cure was found to be perfect—union seeming to have taken place by the first intention—only the line of cicatrization remained, showing very plainly the extent of the injury, and its relation to the fistulous opening.

This difference in the results of nature to repair its injuries, presenting the same characters, influenced by the same causes, is of no practical importance in the present instance, yet it is a fact none the less interesting.

As to the operation, two modes of procedure very naturally suggested themselves: one was to close the fistula and afterward the cleft; the other was to complete the whole under one operation. The latter, though more difficult of execution, was the one I adopted, for the reason that the patient would not require to be confined so long.

A reparation of the cervix I did not consider essential to the success of curing the fistula: still I determined to effect it, if possible, in view of the probable results of parturition, should it occur again. With such a deep fissure existing in the anterior lip of the cervix, it can very readily be perceived how much more liable this point would be to give way under powerful uterine contractions, attended by a reproduction of the fistula, and perhaps laceration of the uterus itself, with all their dreadful consequences, as dribbling of the urine, metritis, peritonitis, etc.

On the eighth day after admission, assisted by Dr. Clanton and Mr. Duncan, a medical student, I proceeded to operate. The method employed was that of Dr. J. Marion Sims, (who, be it to the honor of America, yea, of the world, has accomplished more in this hitherto difficult branch of the practice than any surgeon living.)

A detailed account of the different steps of the operation I shall purposely omit, as it would be tedious and perhaps uninteresting; therefore, only a single feature of it will be presented—the application of the suture apparatus. From my description of the injury, it will now be seen that the clamps had to be applied with all the accuracy observed under ordinary circumstances to parts totally different in structure and function, with a view of obtaining, by a natural and common process, a natural and common result—union by the first intention.

---

* New Orleans Medical and Surgical Journal. May No., 1854.

The dense, strong and unyielding tissue of the cervix had to be made to harmonize with the erectile, spongy and elastic tissue of the vagina. In lodging the wire sutures in their respective places, great care had to be taken that the needle was entered and brought out at a distance from the fistula and cleft, corresponding to the extent of elasticity or adaptation of the parts. If too far removed, there was danger of the edges becoming everted, and *vice versa.* In neither instance could coaptation be effected—a desideratum indispensable to success. The difficulties and perplexities of such a task, simple as its execution may appear, can scarcely be realized by one who has never attempted it. Having, then, thoroughly freshened and shaped the edges of the injured parts, the first suture was carried through the cervix in its lower third; the second was entered on a line a little exterior to the first—carried across the upper extremity of the fistula, and out at a corresponding point at the opposite side; the other two were entered in a similar manner at equal distances below. To theends of the sutures, on the right side, a clamp was fixed and made to take its proper place, the upper end resting against the side of the cervix. The one on the opposite side was arranged in a similar manner, with the proximal ends of the sutures passing through it. Traction being now made upon these ends, the edges of both fistula and cleft were brought in direct opposition. In this relation they were maintained by compressing a small shot on each suture close to the clamp; after which the sutures were cut off close to the shot, and the patient put to bed. The self-retaining catheter was next introduced into the bladder, and the operation then completed.

On the day following menstruation came on, and soon afterward I noticed a bloody state of the urine, and slight leakage of the bladder. This condition of things continued four or five days, or till the catamenia ceased when all unfavorable indications disappeared, and the patient seemed to do well. That a small opening in the bladder existed during this period, to allow of leakage and commingling of the two fluids, there can be no doubt. The most remarkable thing about it is, that it should have closed after so long a time.

The explanation of the result, I think, is this: A small groove remained at the bottom of the cervical cleft, after the application of the clamps, thus admitting a portion of the catamenia to pass along to the upper extremity of the fistula when it entered the bladder. Owing to the peculiar situation of the opening, and its valve-like form, only a very slight leakage could take place, and this, I imagine, as a result of some effort or change of position on the part of the patient. The urine, then, having little or no tendency to escape here, and the edges of the opening being still in a freshened state, union of the parts followed immediately upon the cessation of the catamenial flow.

On the fifteenth day of the operation I removed the clamps, when union at all points seemed to be perfect. A small notch at the extremity of the anterior lip of the cervix was the only evidence of the deep cleft which had existed there. The patient was now allowed to get up. At first she could not retain her urine longer than two or three hours without some pain in the region of of the bladder. This difficulty, however, gradually diminished as the organ regained its natural tone, which it did in a few days. —*Southern Med. Jour.*

MONTGOMERY, May 10, 1855.

